# Periscope

**Published:** 1882-04

**Authors:** 


### PERISCOPE.

N. A. JOURNAL OF HOMŒOPATHY: February. Dr. Skinner gives, as symptoms of *Natrum bicarbonicum:* "Slight giddiness, accompanied with a dirling-tingling in my *right* thumb, fore and middle fingers, whilst holding the pen; the whole *right* arm felt at the same time as if it were about to become paralyzed." These symptoms lasted a few minutes; returned again when empty-handed. Some interesting "cases from practice" are translated, with excellent comments, by "S. L." Thus a case of metrorrhagia after abortion is narrated, in which *Crocus* and *Sabina,* carelessly prescribed, failed to relieve the hemorrhage. *Ipecac.* was then given and relieved. The physician prescribing, Dr. Kaeseman, remarks: "After a few doses the pain (abdominal pain with cutting) and vomiting ceased; the hemorrhage became trifling. * * * *Aude sapere,* dare to heal such fearful cases according to the strict selection of the simillimum, and you may throw to the dogs all adjuvants and palliatives." We may here remark that the *Ipecac.* hemorrhage is a *steady* flow of *bright* red blood; the patient complains of continued nausea, which vomiting does *not* relieve (*Sanguin.*). Sometimes cannot vomit, though desire to do so is great. A case of Diabetes Mellitus is narrated by Dr. Skinner, in which the following mental symptom was present: *Patient often felt as if he could* kill without hesitation any one who *offended him;* only he knew better than to do it." For this *Hepar s.c.* was advised by Dr. Berridge. Seven days after commencing the *Hepar,* all "trace of sugar, for the first time in one year," disappeared, and urea again appeared in the urine. A hard, dry "winter cough," for which *Sulph., Bryonia* and *Nux* were tried in vain, at last yielded to *Lycopodium;* which drug was chosen, because the patient was known to be of a "*miserly disposition.*"

Dr. T. F. Allen continues his excellent "critical examination of our materia medica:" considering *Aloes.* This series of articles are of great value, and do even greater credit to their author than the original work. Many can build, but very few can see flaws in their own work.

HOM. JOURNAL OF OBSTETRICS. February. The editor, Doctor Minton, gives the following repertory of head symptoms:

BEFORE MENSTRUATION.

HEADACHE.—Pain in the head: *Acon., Act. rac., Alum., Bell., Bor., Brom., Bry., Cal. c., Calc. phos., Carbo a., Carbo v., Cinnab., Cup., Gels., Glon., Hepar s., Hyper., Iod., Kreos., Lach., Nat. c., Nat. m., Nit. ac., Nux v., Plat., Puls., Sulph., Verat. a., Zinc.*

——, anxiety, with: *Carbo a.*

——, ceases temporarily when the flow commences: *Alum., Lach., Verat. a.*

——, contractive: *Hepar s.*

——, dull: *Hyper.*

——, evening, in the: *Zinc.*

——, eyes, just above: *Bell., Hyper., Silic., Xan.*

——, forehead, in the: *Acon., Brom., Silic.*

——, heavy: *Act. rac.*

——, periodical: *Ars.*

——, pressive: *Act. rac.*

——, semilateral: *Calc. p., Cinnab., Puls.*

——, sick: *Gels.*

——, sleep, on awakening from: *Alum., Lach.*

——, stinging: *Ferr.*

——, stitching—left side: *Calc. p.*

——, throbbing: *Bell., Bor., Glon., Lach., Petrol.*

——, relieved by external pressure: *Bell.*

——, top of head would come off, as if: *Xan.*

——, beating in: *Bor.*

——, buzzing in: *Kreos.*

——, cannot raise it from the pillow on account of the pain: *Cinnab.*

——, congestion of blood to the: *Acon., Apis, Bell., Bry., Cup., Gels., Glon., Hepar s., Hyper., Merc., Trill.*

——, heat in: *Apis, Bell., Calc. c., Con., Ignat., Iod., Ipec., Petrol., Thuja.*

——, —— — and heaviness: *Ignat.*

——, —— —, running to top of: *Iod.*

——, —— —, to back part: *Bell., Sepia.*

——, heaviness in: *Ignat.*

——. humming in: *Kreos.*

——, pain in, with sensation as if the eyes would fall out: *Brom.*

——, pressing in: *Petrol.*

——, sides of, pain in: *Calc. p., Cinnab., Puls.*

——, — — — —, left: *Calc. p.*

——, stinging in: *Ferr.*

——, stitches in; *Calc. p.*

——, throbbing in: *Bell., Bor., Glon., Lach., Petrol., Gels., Sulph.*

——, — — relieved by external pressure: *Bell.*

VERTIGO: *Acon., Bor., Bry., Calc. c., Con., Lach., Nux m., Phos., Puls., Verat. a*

——, on rising up: *Acon., Bry.*

——, —— turning over in bed: *Con.*

——, when going down stairs: *Bor.*

VERTIGO: when going up stairs: *Calc. c.*
——, —— lying down: *Con.*
——, with drowsiness: *Ant. t.*
——, —— eructations: *Puls.*
——, —— headache: *Lach.*
——, —— palpitation: *Alum.*

DURING MENSTRUATION.

HEADACHE:—Pain in the head: *Acon., Agar., Aloe, Alum., Amm. c., Amm. m., Ant. c., Apis, Argt., Ars., Bell., Berb., Borax, Bovis, Brom., Bry., Bufo, Calc. c., Carb. v., Castor, Cham., Chin., Cicut., Cocc., Con., Cub., Cura., Cycl., Euph., Ferr., Gels., Glon., Graph., Hyos., Ignat., Kali b., Kali c., Kreos., Lach., Laur., Lyco, Mag. c., Mag. m., Mag. s., Nat. c., Nat. m., Nux v., Phos., Plat., Puls., Rhod., Sang., Sepia, Silic., Stan., Sulph., Verat. a., Xan., Zinc.*
——, after breakfast: *Lyc.*
——, —— noon, *Nux v.*
——, aggravated by coughing: *Nat. m.*
——, —— ——, mental excitement: *Nux v.*
——, —— ——, motion: *Bry., Glon., Nux v.*
——, —— ——, noise: *Bell., Nux v.*
——, —— ——, sneezing: *Nat. m.*
——, ——, while in the open air: *Nux v.*
——, —— ——, lying down: *Nux v., Puls.*
——, alternating with pain in the back: *Aloe.*
——, ameliorated at night: *Mag. c.*
——, —— by bathing in cold water: *Aloe, Ars.*
——, —— —— pressure: *Bell., Puls.*
——, as if the brain were torn in pieces: *Coff.*
——, — — — eyes would fall out: *Brom.*
——, — — — head would split: *Bry., Nat. m., Sepia.*
——, awakens with: *Agar., Lach., Nat. m.*
——, —— —— at night: *Agar.*
——, beginning in the occiput and spreading to the top of the head: *Calc. c.*
——, —— —— —— —— spreads upwards and settles over the right eye: *Sang.*
——, boring: *Calc. c., Sepia.*
——, bruised pains back of the orbits: *Gels.*
——, bursting: *Bry., Calc. c., Glon., Lyco., Nat. m., Sepia, Sang.*
——, comes on gradually and as gradually subsides: *Arnica, Plat., Stann.*
——, —— — suddenly and suddenly subsides: *Bell.*
——, congestive. See Head, congestion of.
——, continuous: *Carb. v., Gels., Hepar s., Rhod.*
——, contractive: *Carb. v., Lyco.*
——, distressing: *Xan.*
——, drawing in lines from the neck up over head: *Sang.*
——, dull: *Act. rac., Gels., Hyper., Lyco., Ust.*

HEADACHE, dull, with stupor: *Lyco.*
——, ——, —— vertigo: *Lyco.*
——, extending to face and teeth: *Puls.*
——, —— —— the occiput: *Bell.*, *Calc. c.*, *Sepia.*
——, —— from occiput to right orbital region: *Sang.*
——, extended from the occiput to the top of the head: *Calc. c.*
——, flow becomes checked, when the: *Acon.*, *Alum.*
——, followed by heat and dryness of the lips: *Nux v.*
——, frontal. *Aes. hip.*, *Amm. c.*, *Bell.*, *Brom.*, *Bry.*, *Castor*, *Kali b.*, *Nux v.*, *Sang.*, *Sepia*, *Sulphur.*
——, —— as if everything would fall out at the forehead, when stooping: *Bry.*
——, ——, —— the eyes would fall out: *Brom.*
——, ——, —— — — — be pushed out: *Nux v.*, *Sang.*
——, ——, —— it would burst: *Bry.*, *Nux v.*
——, ——, constrictive, as from a band about the forehead: *Gels.*, *Helon.*, *Iod.*, *Merc.*, *Plat.*
——, ——, lacerating: *Castor*, *Phos.*, *Rat.*
——, ——, over the eyes: *Act. rac.*, *Graph.*, *Sang.*
——, ——, —— —— right eye: *Sang.*
——, ——, pain begins in the occiput, spreads upwards, and settles over the right eye: *Sang.*
——, —— pressing: *Bell.*, *Bry.*, *Cact.*, *Cycl.*, *Mag. c.*, *Nat. m.*, *Sepia*, *Silic.*, *Sulph.*
——, ——, pulling: *Mag. c.*
——, ——, shooting: *Rat.*
——, ——, splitting: *Nat. m.*
——, ——, transient: *Rat.*
——, girls', school: *Nat. m.*
——, gradually increasing to its highest point, then as gradually declining: *Arnica*, *Plat.*, *Stan.*
——, morning: *Bry.*, *Graph.*, *Kali c.*, *Lach.*, *Nat. m.*, *Nux v.*, *Verat.*
——, ——, on awaking: *Lach.*, *Nat. m.*
——, ——, till evening: *Kali c.*
——, nervous: *Act. rac.*, *Bry.*, *Calc. c.*, *Coloc.*, *Gels.*, *Puls.*, *Verat.*
——, ——, especially over the right eye: *Sang.*
——, neuralgic: *Gels.*, *Kalm.*, *Sang.*
——, night, at: *Laur.*, *Mag. c.*
——, ——, toward: *Graph.*
——, over the eyes: *Lach.*, *Sang.*
——, —— — nose, root of: *Arnic.*, *Hepar*, *Ignat.*, *Lach.*
——, pain gradually increases and as gradually decreases: *Arnic.*, *Plat.*, *Stan.*
——, pressive: *Acon.*, *Act. rac.*, *Nat. m.*, *Nux v.*
——, ——, from within outward: *Kreos.*
——, ——, —— —— —— in both temples: *Bry.*
——, semilateral: *Ars.*, *Calc. c.*, *Chin.*. *Cicuta.*, *Colch.*, *Nux v.*, *Puls.*, *Sepia*, *Verat. a.*

HEADACHE, sick: *Gels., Phos., Puls., Nux v., Sang.*
——, ——, right side, with a sensation as if the eyes were being pushed out: *Sang.*
——, splitting: *Bry., Glon.*
——, stinging: *Acon., Mang.*
——, stupefying: *Calc. c., Cina, Lyc.*
——, tearing: *Calc. c., Nat. c.*
——, temples, pain in, as if they were squeezed together: *Lyco.*
——, ——, —— —— —— —— a nail were driven out through the sides of the head: *Ignatia.*
——, ——, —— bursting: *Glon. Lach.*
——, ——, pains in contractive: *Lyco.,*
——, ——, neuralgic: *Lob.*
——, ——, —— —— stitching, left side: *Calc. p.*
——, throbbing: *Acon., Bell., Borax, Bry., Cact., Calc., c., Chin., Glon., Ignat., Lach., Mag. c., Nat. c., Puls., Sang.*
——, —— rushing in the ears, with: *Bor.*
——, ——, toothache, with: *Lach.*
——, when the flow is checked: *Acon., Alum.*
——, with buzzing and humming: *Bor., Kreos.*
——, —— chilliness: *Eupion.*
——, —— chattering of the teeth: *Nux. v.*
——, —— colicky pains: *Carb. v.*
——, —— debility, excessive: *Nux v.*
——, —— eructations: *Graph.*
——, —— fainting: *Graph., Nat. m.*
——, —— —— in the morning: *Graph.*
——, —— heat of the head: *Ignat., Mag. c., Nat. m.*
——, —— heaviness of the head: *Ignat., Kali c., Mag. c., Mag. s., Nat. m.*
——, —— internal heat: *Nat. m,*
——, —— nausea: *Graph., Ipec., Nat. m., Phos., Puls., Sang., Verat. a.*
——, —— pain extending to the ears: *Puls.*
——, —— —— in the back: *Aloe.*
——, —— —— —— —— face: *Puls.*
——, —— —— —— —— teeth: *Calc. c., Graph., Lach., Merc., Puls.*
——, —— stupor: *Ham.*
HEAD—band, or tape about, sensation of: *Gels., Helon., Iod., Merc., Plat.*
——, beating in. See Headache, throbbing.
——, buzzing in: *Brom., Kreos.*
——, coldness in and about: *Calc. c.*
——, —— on the vertex: *Sepia, Sulph., Verat. a.*
——, —— —— —— —— as from ice: *Verat. a.*
——, confused, or crowded feeling in the: *Cocc.*
——, congestion of the: *Acon., Apis, Bell., Bry, Cact., Calc. c., Caust., Chin., Con., Elaps, Gels., Glon., Merc., Mosc., Nat. m., Nux v., Phos., Sang., Sulph., Verat.*
——, dizziness of. See Vertigo.

HEAD, enlarged, feels: *Argt., Glon.*
——, forehead, perspiration on: *Phos., Verat. a.*
——, —— pains in. See Headache.
——, full of absurd ideas: *Stram., Verat. a.*
——, —— feels too: *Argt., Bell., Calc. c., Eupion, Glon.*
——, heat in, *Apis, Arnic., Bell., Calc. c., Carb. a., Caust., Ignat., Ipec., Kali b., Lycop., Mag. c., Mag. s.*
——, —— —— with cool extremities: *Arnic.*
——, heaviness of: *Calc. c. Ignat., Kali c., Mag. c., Mag. s.*
——, —— and vertigo: *Calc. c.*
——, humming in: *Brom., Kreos.*
——, —— —— increased by motion: *Kreos.*
——, large, feels too: *Argt., Glon.*
——, movements of, twitching, jerking, etc.: *Cicut.*
——, numbness in the brain, sensation of:
——, pains in, as if pierced by a nail: *Arnic., Ignat., Nux v.*
——, —— —— coming on gradually and as gradually declining: *Arnic., Plat., Stan.*
——, —— —, —— —, suddenly and suddenly subsiding: *Bell.*
——, perspiration on: *Cham., Merc., Phos., Verat.*
——, pressure in: *Cycl., Sepia.*
——, —— —, fore part of: *Bell., Bry., Eupion, Sepia, Stan., Sulph.*
——, —— —, from within outward: *Kreos.*
——, pulsating in, painless: *Eupion.*
——, rush of blood to. See Head, Congestion of.
——, stupefaction of: *Cycl., Ham.*
——, swaying in: *Cub.*
——, top of—vertex—burning in: *Lach., Phos., Sulph.*
——, —— —, coldness on: *Verat. a.*
——, —— —, heat in: *Sulph.*
——, —— —, pain in, as if a nail were driven into: *Nux v.*
——, —— —, tearing in, at night: *Laur.*
——, —— —, pressure on: *Calc. c., Castor., Nux v.*
——, tightness in: *Lyc., Nat. m., Phos.*
——, trembling of: *Ant. c., Cicut., Cannab., Cub.*
VERTIGO: *Acon., Amm. c., Ant. t., Argt., Bor., Brom., Cact., Calc. c., Carb. v., Caul., Caust., Con., Cub., Cycl., Elaps, Ferr., Gel., Graph., Iod., Kali b., Lach., Lyco., Mosc., Nux v., Phos., Plat., Puls., Sulph., Thuj., Trill., Ust.*
——, frequent attacks of: *Plat.*
——, in the morning when rising: *Acon., Caul., Cocc., Con., Graph., Lach., Mag. c., Nat. m., Nux v., Rhus, Sepia, Sulph.*
——, on ascending a height, or going upstairs: *Bor., Calc. c., Con., Phos., Sulph.*
——, — descending or going downstairs: *Bor., Ferr., Stan.*
——, — rising from a recumbent position: *Acon.*
——, — ——— ——— — sitting position: *Calc. c., Sulph.*
——, when looking at running water: *Brom.*
——, ——— lying down: *Apis, Cham., Con., Nux v., Rhus, Thuj.*

VERTIGO: when lying down, an attempt is made to turn over: *Con.*
——, ——— moving the head: *Cocc., Con., Ipec., Glon., Mosc.*
——, ——— rising from a stooping position: *Bell., Calc. c., Nat. m.*
——, ——— turning the head in bed: *Con.*
——, ——— walking: *Apis, Ars., Bell., Calc. c., Con., Ipec., Kali c., Lyc., Nux v., Pet., Phos., Puls., Sepia, Zinc.*
——, with headache: *Caust., Cub., Cycl., Kali b., Phos., Sulph.*

AFTER MENSTRUATION.

HEADACHE.—*Agar., Carb. ac., Kali b., Lach., Lyco., Nat. m., Puls.*
——, and dizziness: *Agar.*
——, nervous: *Carb. ac.*
——. recurring at short intervals: *Lyco.*
——, stinging: *Berb.*
——, stitching: *Lyco., Nat. m.*
——, —— in the forehead, with stupor: *Lyco.*
——, throbbing: *Calc p., Carb. a., Glon.*
HEAD, congestion of: *Chin., Nat. m., Sulph., Thuj.*
——, dullness in: *Nat. m.*
——, heaviness in: *Nat. m.*
——, pains in as if the top of the head were being lifted off: *Ust.*
——, —— —— pressive: *Ust.*
——, —— —— stinging: *Berb., Nat. m., Plat.*
——, —— —— stitching, returning at short intervals: *Plat.*
——, soreness of: *Plat.*
VERTIGO.—*Agar., Ant. t., Con., Puls., Ust.*

